# TFE3 nuclear expression as a novel biomarker of ovarian sclerosing stromal tumors and associated with its histological morphology

**DOI:** 10.1186/s13048-023-01241-y

**Published:** 2023-08-01

**Authors:** Li Zhao, Zhongfeng Yang, Yan Zhou, Yuping Liu, Qiuping Luo, Qingping Jiang, Hui Wang, Na Wang

**Affiliations:** 1 The Third Affiliated Hospital of Guangzhou Medical University, Guangzhou, 510150 China; 2 Guangdong Provincical Key Laboratory for Major Obstetric Diseases, Guangzhou, 510150 China; 3 Affiliated Cancer Hospital & Institute of Guangzhou Medical University, Guangzhou, 510000 China; 4 The Fourth Affiliated Hospital of Guangzhou Medical University, Guangzhou, 510150 China

**Keywords:** Ovarian sclerosing stromal tumors, TFE3, Novel biomarker

## Abstract

Sclerosing stromal tumors of the ovary are benign and tend to occur in youthful women with lobular structures at low frequencies. Three types of cells, including luteinized cells, short spindle myoid cells, and intermediate cells, are found in the lobules which abundant in the blood vessels. Currently, immunohistochemistry is used to detect normal follicles, sclerosing stromal tumors, granulosa cell tumors, and fibromas/thecomas. Our research results showed that transcription factor enhancer 3 (TFE3) was moderate to strong positive in the theca interna layer of normal follicles. TFE3 was expressed in seven out of eight sclerosing stromal tumors, mainly in luteinized cells. It did not express in 20 granulosa cell tumors. Of the nine fibromas/thecomas, TFE3 was weakly staining in 2 cases and negative in the remaining 7 cases. The expression of TFE3 was also weak in only one microcystic stromal tumor. 8 cases of sclerosing stromal tumors were analyzed by FISH using a TFE3 separation probe, and the results were negative. In short, as a nuclear transcription protein, TFE3 specifically expressed in sclerosing stromal tumors and could serve as a new marker for the diagnosis and differential diagnosis of sclerosing stromal tumors. Moreover, we speculate that TFE3 will promotes the formation of the vascular plexus after entry into the nucleus, which can further explain why sclerosing stromal tumors are different from other ovary sex-cord stromal tumors.

**Supplementary Information:**

The online version contains supplementary material available at 10.1186/s13048-023-01241-y.

## Introduction

Ovarian sex-cord stromal tumors, also known as gonadal stromal tumors, includes tumors that arise from gonadal and stromal cells such as granulosa cells, thecoma cells, fibroblast cells, sertoli cells, or Leydig cells. These tumors are made up of the cells mentioned above alone or in combination. As the most common sex-cord stromal tumor, fibroma consists of spindle cells and varying amounts of collagen with or without thecoma cells, and account for about 4% of ovary tumor, while thecoma account for 1/3 of granulosa cell tumor. Fibromas/thecomas are mostly seen in middle-aged and older people, with less than 10 percent of those younger than 30. Granulosa cell tumor can be divided into juvenile granulosa cell tumor and adult granulosa cell tumor based on their morphology. Adult granulosa cell tumors account for 1 percent of ovarian tumors and are more common in postmenopausal women. Classical histology of granulosa cell tumor is diffuse or nested growth of tumor cells, mononuclear and coffee-bean-like nuclei, and the formation of Call-Exner bodies in 50% of tumors. Microcystic stromal tumor is a rare subtype recently reported as stromal tumor [1], and patients ranged in age from 23–71. The Microcystic stromal tumor is typically characterized by a varying number of small cysts, which are often extremely prominent and fuse with each other to form the distinctive morphological features of the tumor.

Sclerosing stromal tumor (SST) is a rare sex cord-stromal ovarian tumor that was first reported by Chalvaridjian and Scully in 1973 [2]. SST occurs predominantly in young women of 20–30 years of age [3, 4], and its clinical symptoms include pelvic pain, menstrual irregularity [5], and nonspecific symptoms associated with ovarian cysts. In a few cases, patients had elevated serum levels of CA125 [6]. However, the levels of hormones in these patients were not affected. Focal adenoid hyperplasia was diagnosed in only 1 of the 10 cases, but endometrial biopsy could not be performed in this case [6]. In addition, a case of ovarian SST complicated by endometrial adenocarcinoma has been reported [7].

The characteristic feature of SST is the abundance of blood vessels in the nodule. Computed tomography (CT) and magnetic resonance imaging (MRI) have been employed to diagnose SSTs, particularly to assess their vascularization [8–10]. New ultrasound technologies have been developed to facilitate the exploration of adnexal masses, such as the diagnosis of tissue vascularization via colour Doppler [11]. This blood flow feature of SST is relatively unique, which is helpful to differentiate from other sex cord stromal tumors and ovarian malignant tumors. This method not only obtains examination results quickly but also reduces the economic burden on patients.

In general, SST is unilateral, mostly 5–10 cm, with well-defined boundaries and sometimes a thin fibrous envelope. It is a grey‒white to grey‒yellow nodular oedema, and unilocular cystic cases are rare [12]. According to previous studies, SST consists of three types of cells, lipid-rich cells, fibroblast-like cells, and undifferentiated stromal mesenchymal cells, with intermediate morphology showing different degrees of differentiation [13]. In addition, some studies indicate that tumor cells in SST have the characteristics of muscle-like cells and express SMA or desmin [14, 15]. Growing evidence shows that cytoplasm-rich cells express calretinin and inhibin [16] but do not express SMA, desmin, CK, and CK7 [17–19]. In some cases, lipid-rich cells express CD10 [20] and Melan A [21, 22]. These cells are similar to normal cells in the ovary but have not been clearly defined.

One study found that TFE3 was highly expressed in sclerosing stromal tumors and noted that TFE3 was highly expressed in the nucleus of lutein cells and polygonal-to-round tumor cells in 7 out of 9 patients with SST, however, neither luteinized fibromas nor thecomas express appreciable levels of TFE3 [23]. Fluorescence in situ hybridization (FISH) analysis revealed the presence of trisomy 12 in > 20% of SST cells [17]. In 2020, using whole-exome, targeted capture, and RNA sequencing, Sarah et al. reported that 65% (17/26) of SST patients had recurrent *FHL2-GLI2* fusion genes and that 15% (4/26) had other *GLI2* rearrangements [24]. Specifically, these genetic abnormalities were not detected in other types of sex cord-stromal tumors (*n* = 48) and common cancers (*n* = 9,950).

In this study, the origin of SSTs was discussed. TFE3 immunohistochemical and molecular analyses were performed on SSTs and other types of ovarian sex cord-stromal tumors. The mechanism for the entry of TFE3 into the nucleus and the morphological changes of SST related to that were discussed.

## Materials and methods

### Patients

The records of 38 patients who had undergone surgical resection of sex cord-stromal ovarian tumors at The Third Affiliated Hospital of Guangzhou Medical University (Guangzhou, China) between December 2013 and December 2021 were reviewed. The focus of this study is sclerosing stromal tumor (8 cases), and its main differential diagnoses, which include thecoma/fibroma (9 cases), granulosa cell tumor (20 cases), and microcystic stromal tumor (1 case) in turn. The age of the patients ranged from 17 to 70 years, and they had not received any preoperative therapy.

Ethical approval was obtained from the institutional review board of the ethics committee.

### Lipid stains (Oil Red O)

The biopsy samples were placed in a tissue tek container (Sakura Finetek, CA, USA) and then filled with tissue tek OCT compound gel. After being cut into 7-µm slices, the samples were snap-frozen in liquid nitrogen and stained with Oil Red O according to standard procedures.

The oil red O fat staining method is usually used to detect fat in tissues or cells. Oil red O is a fat-soluble dye that is a strong fat solvent and fat dye and can be highly dissolved in fat. Its dyeing principle is that oil red O can specifically adsorb with the neutral triglycerides, lipids and lipoproteins in tissues and cells to make fat dye. The solubility of dye in intracellular lipids is greater than that in solution.

### Immunohistochemistry (IHC)

As the experimental subjects, tumors or normal tissues were collected after paraffin removal. Consecutive 4-μm-thick unstained sections were used for immunohistochemical staining, which was performed using the Leica automatic immunostaining device (Leica Microsystems, Inc.). Primary antibodies against CD10 (1:100; no. 563871; DAKO; DK), α-inhibin (1:100; no. GT230202; CHN), SMA (1:50; no. MAB-0980; MXB; CHN), desmin (1:300; no. GT225202; Gene tech; CHN), TFE3 (1:100; no. ZA-0657; ZSGB-BIO; CHN), calretinin (1:100; no. ZM-0063; ZSGB-BIO; CHN), WT-1 (1:100; no. ZM-0269; ZSGB-BIO; CHN), and EMA (1:300; no. GM061302; Gene tech; CHN). Appropriate positive and negative controls were simultaneously stained to validate the staining method.

### Immunohistochemistry was conducted according to previously described methods

All slides were reviewed and scored independently by three pathologists. The pathologists were blinded to the experiment. The scoring method was based on the intensity (0, no staining; 1 + , weak staining; 2 + , moderate staining; 3 + , strong staining). Tumors scored as positive for TFE3 demonstrated moderate (2 +) and strong (3 +) nuclear immunoreactivity, and negative for TFE3 demonstrated weak (1 +) and no nuclear immunoreactivity [25].

### Fluorescence in situ hybridization (FISH) analysis

The *TFE3* isolation probe was provided by Guangzhou LBP Medicine Science and Technology Co., Ltd. (China). Specific operations were performed according to the manufacturer’s protocol. The results showed that there was no fracture of the* TFE3* gene in females (2 yellow) and males (1 yellow). Additionally, 1 red 1 green 1 yellow in females and 1 red 1 green in males indicated that *TFE3* had a balanced translocation and that the gene was fused, while 1 red 2 yellow in females and 1 red 1 yellow in males showed an unbalanced translocation and a fracture of the *TFE3* gene.

### Statistical analysis

The statistical analysis was performed using SPSS 19.0 software (SPSS, USA). The χ2 test was used to estimate the correlation between the expression of TFE3 and sex cord-stromal tumors. A cumulative survival A probability value of 0.05 or less was considered significant.

## Results

### Clinical findings

As shown in Table 1, the age range of the patients with SSTs was 17-39 years. The levels of hormones were normal in all patients. However, menstruation was irregular in cases 5 and 7. In case 5, an adnexal mass was found during physical examination, and no abnormality was found in the endometrium. In case 7, endometrial biopsy showed atypical hyperplasia, and an adnexal mass was found. In case 4, in which the patient presented with abdominal distension for 1 year, an adnexal tumor was found on B-ultrasound with peritoneal effusion. Adnexal tumors were found in other patients during physical examinations.

**Table 1:** Clinical features of SSTs

Case	Age (years)	Gross	CA199 (U/ml)	CA125 (U/ml)	Size (mm)	Menstruation	Endometrium	Management	Outcome Period	Follow-up (months)
1	23	cystic	2	36.4	60	normal	normal	UO	NED	19
2	17	solid	4.83	457.8↑	14	normal	normal	UO	NED	58
3	26	solid	ND	ND	160	normal	normal	UO	NED	84
4	26	solid	ND	ND	45	normal	normal	UO	NED	82
5	39	solid	< 2	32.2	80	irregular	normal	UO	NED	79
6	29	solid	ND	ND	60	normal	normal	UO	NED	52
7	33	solid	36.82	17.2	50	irregular	atypical hyperlasia	UO	NED	81
8	23	solid	ND	ND	25	normal	normal	UO	NED	52

*SSTs* Sclerosing stromal tumors, *CA 199* Cancer antigen 199, *CA 125* Cancer antigen 125, *ND* no detection, *UO* Unilateral, oophorectomy, *NED* No evidence of disease

### Gross findings

**Fig. 1:**
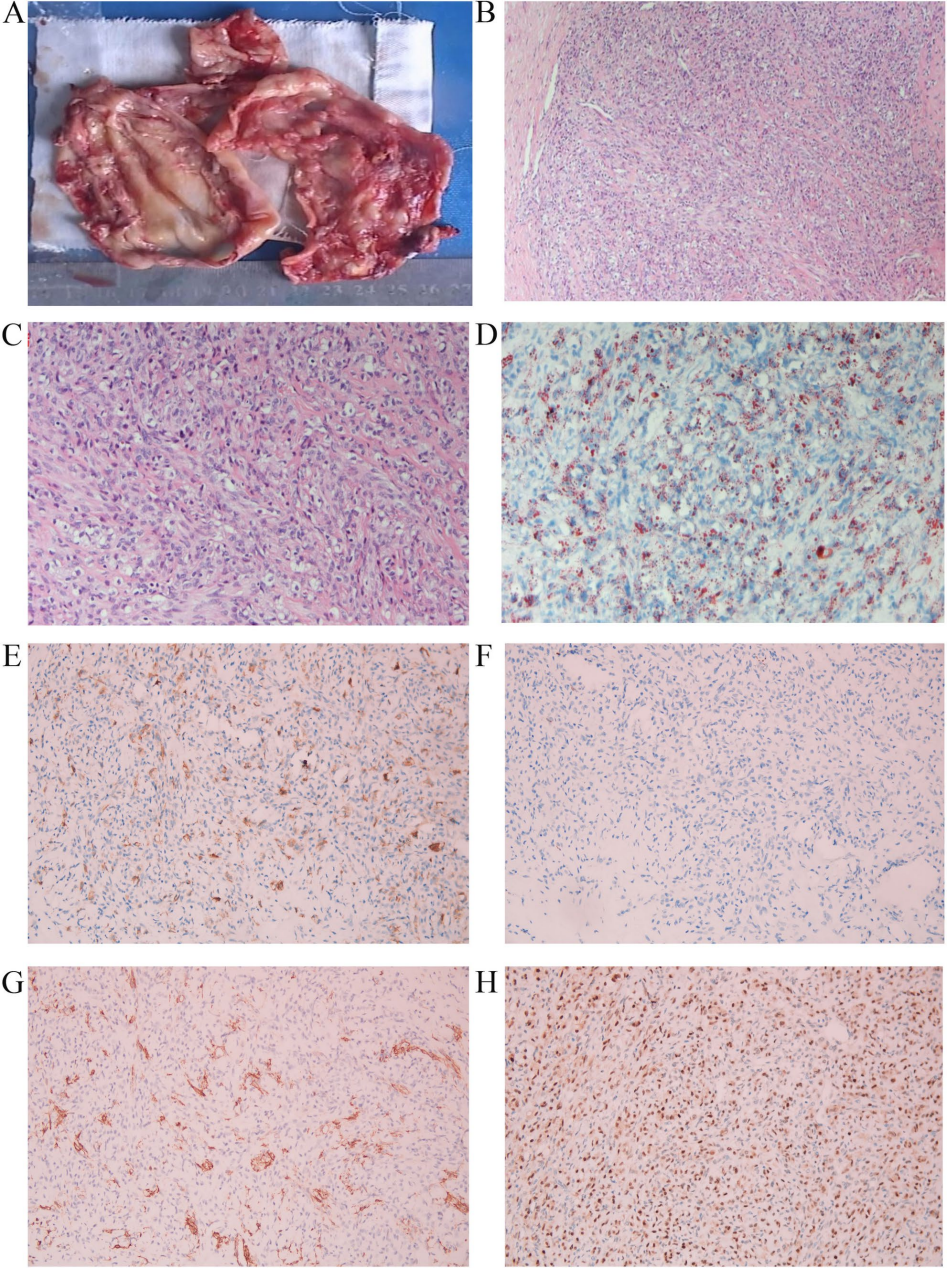
One case of cystic SST. The cut surface of SST was cystic (**A**). The cellular foci consisted of round cells admixed with spindle cells (**B**, × 100 and **C**, × 200). Oil Red O staining was positive in lipid-rich cells (**D**, × 200). The spindle tumor cells weakly expressed SMA (**E**, × 200). The round cells and spindle cells did not express desmin (**F**, × 200). CD34 outlines a rich vascular network (**G**, × 200). TFE3 was diffuse positive in the lipid-rich cells (**H**, × 200)

**Fig. 2:**
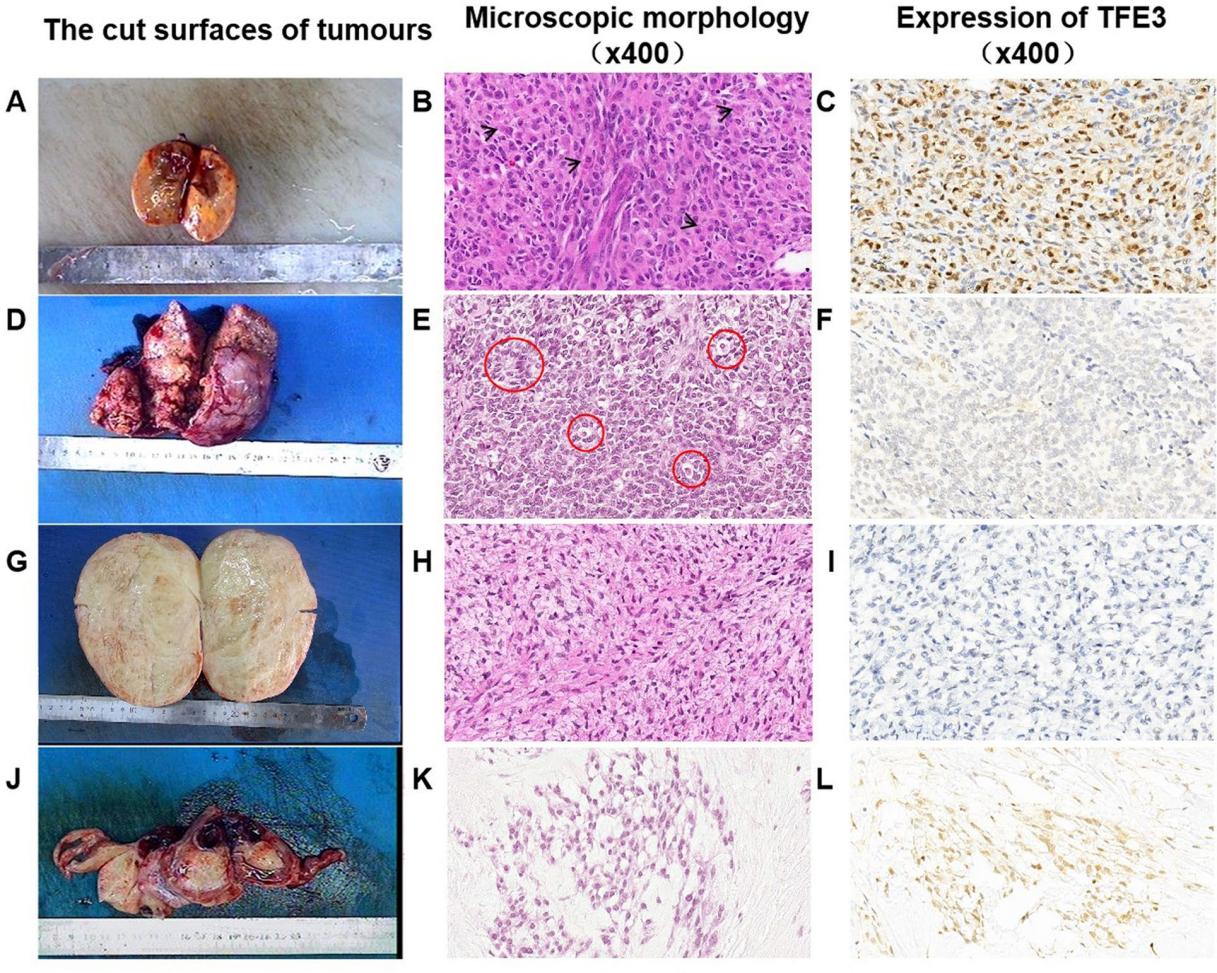
Expression of TFE3 in different types of sex cord stromal tumors. The cut surfaces of SST were solid with yellow (**A**). The cytoplasm of most cells in the lobules was rich, eosinophilic and granular (black arrows) (**B**). Cytoplasm-rich cells express TFE3 (**C**). The cut surface of the adult granulosa cell tumor is nodular and light yellow (**D**). Granulosa cells usually have scanty cytoplasm and pale, uniform, angular to oval, often grooved nuclei that are typically arranged haphazardly to each other, and typical Call-Exner bodies can be seen (red circles) (**E**). TFE3 is not expressed in AGCT (**F**). Thecoma. The typical sectioned surface of a thecoma showing a yellow appearance (**G**). Thecoma. A high-power view shows the characteristic appreciable pale gray cytoplasm, ill-defined cytoplasmic membranes, and scattered collagen bundles (**H**). TFE3 is not expressed in the tumor cells of thecoma (**I**). Microcystic stromal tumor. The sectioned surface showing a solid, yellow appearance (**J**). Microcystic stromal tumor. Characteristic small cysts and hyaline plaques are seen (**K**). The tumor cells do not express TFE3 (**L**)

The sizes of the eight tumors ranged from 14 to 160 mm. All tumors were well-circumscribed nodules, and except for a cystic tumor in one case (Fig. 1A), all tumors were solid (Fig. 2A), ranging from soft to tough. The cut surfaces of the solid tumors were typically white and slightly leafy, with scattered yellow nodules, and were most abundant at the periphery. Focal hemorrhage was observed in one patient. In case 1, the tumor was cystic, with cysts containing thick gelatinous material (Fig. 1A).

### Histologic findings and immunohistochemical results of sclerosing stromal tumors

All tumors, including cystic tumors, showed overt, often discrete, cellular and hypocellular regions, resulting in a pseudolobular appearance (Fig. 1B). The cellular areas and lower cellular intervals were always collagenous, loose collagenous, and markedly edematous. Cellular foci consisted of a mixture of round and spindle cells (Fig. 1C). The cytoplasm of the former was pale, eosinophilic, vacuolated, or foamy to varying degrees (Fig. 1C). Thin-walled, dilated, focally branching (“staghorn”) blood vessels were prominently found in all cases, and markedly conspicuous labyrinthine and hemangiopericytoma-like tumors were evident in some cases (Fig. 2B). Oil Red O staining revealed that the cytoplasm was rich in lipids but not in mucus (Fig. 1D). Spindle cells expressed SMA (Fig. 1E) but not desmin (Fig. 1F). CD34 staining showed abundant blood vessels in the cell lobules (Fig. 1G). Only cells with abundant cytoplasm in the tumors expressed TFE3, and nuclear expression was moderately and strongly positive (Fig. 1H).

### Expression of TFE3 in ovarian sex cord stromal tumors

As shown in Table 2, TFE3 was highly expressed in SSTs (7/8). TFE3 was weakly expressed in two cases of thecoma (2/9) and one case of microcystic stromal tumors (1/1). TFE3 was not expressed in 20 cases of granulosa cell tumors (20/20). We divided all cases into positive and negative TFE3 expression groups [25]. Positive staining for TFE3 demonstrated moderate (2 +) and strong (3 +) nuclear immunoreactivity. Negative results for TFE3 demonstrated weak (1 +) and no nuclear immunoreactivity. The results showed that the positive expression rate of TFE3 in SSTs was significantly higher than that in the other three types of tumors. (*P* < 0.05, Table 2). In the supplementary materials (Fig. S1), we demonstrated the expression of TFE3 by immunohistochemistry in 8 cases of sclerosing stromal tumors. Among them, 5 cases were strongly positive, 2 cases were moderately positive, and 1 case was weakly positive. The intensity and proportion of TFE3 expression were recorded in Table S1. The immunohistochemical results of each TFE3 case have external controls. The negative control was prostate cancer, and the positive control was TFE3 translocation-associated PEComa (Fig. S2).

**Table 2:** Immunohistochemical results of TFE3

Tumor Type	No. of cases	TFE3 IHC				Positive (%)	Negative(%) (%)	χ2	*P* value
		3 +	2 +	1 +	0				
Theca cell tumor and theca fibroma tumor	9	0	0	2	7	0	100	13.39	**< 0.001**
Granulosa cell tumor	20	0	0	0	20	0	100	23.33	**< 0.001**
Microcystic stromal tumor	1	0	0	1	0	0	100	3.94	**0.047**
Sclerosing stromal tumor	8	5	2	1	0	87.5	12.5		

As shown in Fig. 2, TFE3 was expressed mainly in sclerosing stromal tumors (Fig. 2A, B, C) but not in ovarian granulosa cell tumors (Fig. 2D, E, F), thecoma (Fig. 2G, H, I), or microcystic stromal tumors (Fig. 2J, K, L). Moreover, TFE3 was expressed mainly in the nucleus of cells with abundant cytoplasm, and the staining intensity was medium to strong (Fig. 2C).

### Immunophenotypes of theca cells in normal follicles

**Fig. 3:**
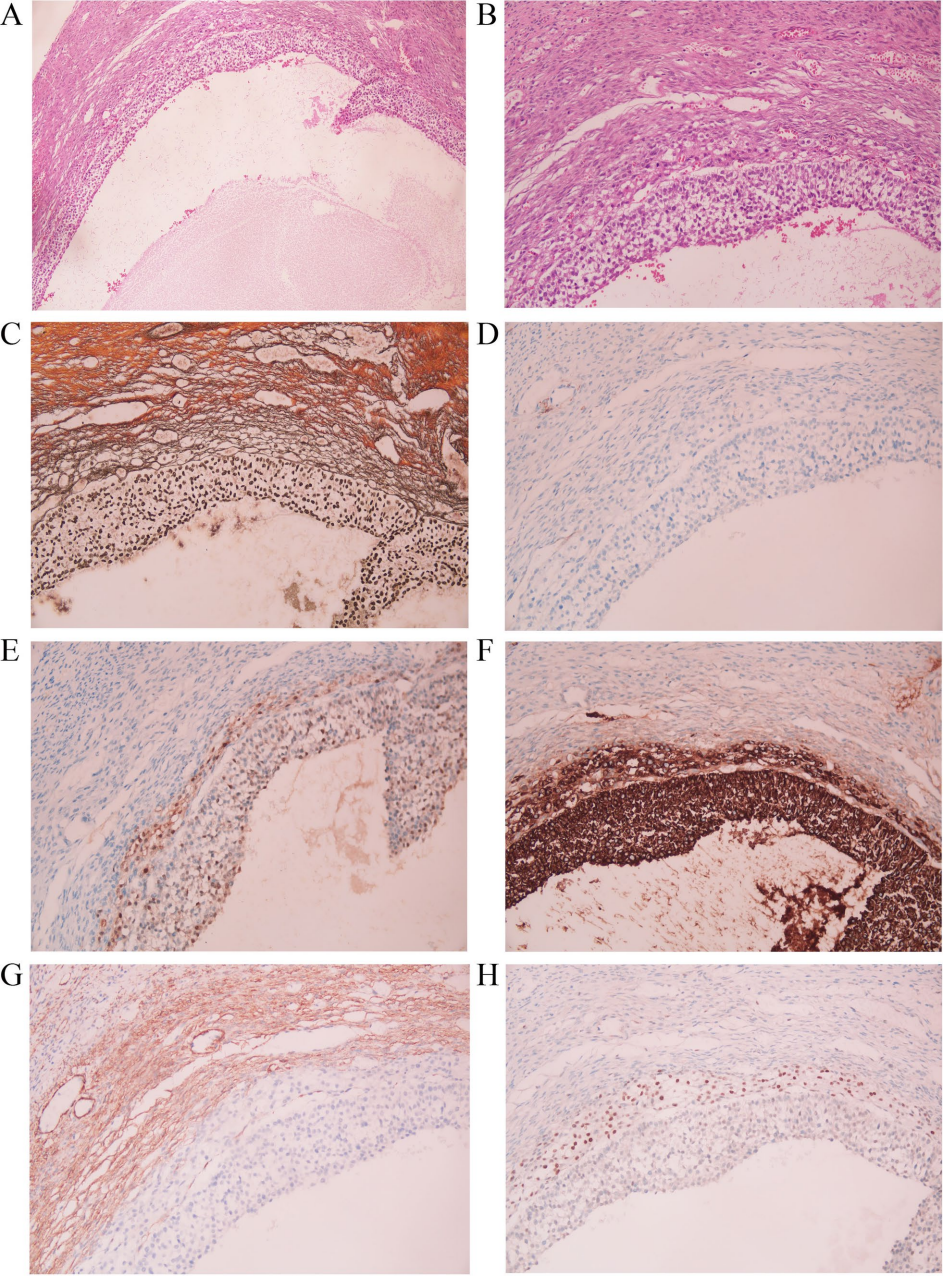
Normal follicular tissue. The follicle was observed at low magnification (× 40) (**A**) and high magnification (× 200) (**B**). Reticular fibers surrounded the inner theca cells and were absent around granulosa cells (**C**). CD10 was negative (**D**). The inner theca cells and granulosa cells expressed calretinin and inhibin (**E** and **F**). SMA was expressed mainly in the outer theca cell layer (**G**). TFE3 was expressed in the inner theca cells (**H**)

No literature has reported the expression mode of TFE3 in normal ovaries. To explore the source of TFE3 immunohistochemistry-positive cells in sclerosing stromal tumors, we collected 10 normal ovarian tissues. Normal ovarian follicles were obtained from specimens removed due to ovarian endometriosis or teratoma. We tested a total of 30 follicles. As shown in Fig. 3, normal follicular structure was observed at low and high magnifications. The boundary between the inner and outer theca layers was not very clear, but the cytoplasm of inner theca cells was rich and clear (Fig. 3A and B). Reticular fibers surrounded the inner theca cells and were absent around granulosa cells (Fig. 3C). CD10 was negative in the inner and outer theca layers (Fig. 3D). The inner theca cells and granulosa cells expressed calretinin and inhibin (Fig. 3E and F). SMA was expressed mainly in the outer theca cell layer (Fig. 3G). A mostly consistent immunophenotype was observed in the rich cytoplasm cells: each demonstrated diffuse TFE3 expression in the inner theca cells but not in the granular layer, outer theca layer, or fibrous tissue in normal follicles (Fig. 3H).

### FISH detection

**Fig. 4:**
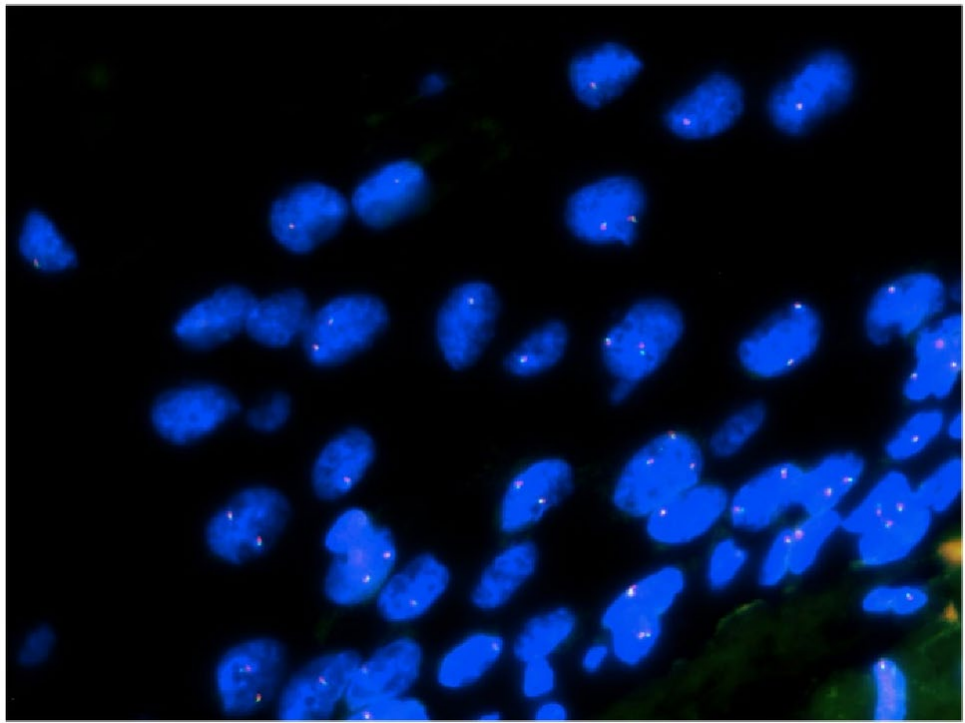
The FISH results. There was no separation of *TFE3* in SST

Using the separation probe of *TFE3*, *TFE3* was detected by FISH in 7 cases of SSTs expressing TFE3. The results showed no separation of *TFE3* in these seven cases (Fig. 4).

## Discussion

*TFE3* is located at Xp11.23, and its protein belongs to the microphthalmia-associated transcription factor (MiTF) family, which plays an important role in the regulation of lysosomal biogenesis and autophagy [26]. The TFE/MiTF family consists of four important members: (i) TFEB, (ii) TFEC, (iii) TFE3, and (iv) MITF [27]. By searching the human protein atlas webtool (https://www.proteinatlas.org/), TFE3 was found to be expressed in adipose tissue, urinary bladder, ovary, testis, and breast, among others. The expression and activity of TFE3 are upregulated in many types of human cancers and are associated with the enhanced proliferation and motility of cancer cells. The main tumors related to TFE3 gene fusion include renal cell carcinoma [28, 29], alveolar soft-part sarcoma (ASPS) [30, 31], epithelioid hemangioendothelioma [32], rare ossifying fibromyxoid tumors [33], malignant chondroid syringoma [34], and perivascular epithelioid cell tumors [35].

Park CK and Kim HS reported that TFE3 was expressed in sclerosing stromal tumors, but there was no abnormality in the *TFE3* gene [23]. As shown in Table 2, our research obtained the same result, namely, that TFE3 was specifically expressed in sclerosing stromal tumors but not in other sex cord-stromal tumors. Moreover, TFE3 was specifically expressed in luteinized cells but not in the other two cell lines.

Sclerosing stromal tumors often occur in young women, and a few cases have the secretion of estrogen and/or androgen. The clinical results of all cases were benign. This study analyzed 8 cases of sclerosing stromal tumors, ranging in age from 17 to 39 years. No hormone abnormalities were found clinically, but 2 patients had irregular menstruation. Follow-up results showed no recurrence. Due to the young age of SST patients and good prognosis, accurate pathological diagnosis is very important to avoid overtreatment. In most cases, we can obtain a positive pathological diagnosis based on the above findings. One aspect of recent emphasis on sclerosing stromal tumors is that typically young people are involved and patients may pregnant. In this case, tumor cells may have more extensive luteinization than usual, which may mask the typical three cell populations [36]. Zhang et al. reported a case of SST in a young woman with atypical cells resembling sarcomas [37]. More specific indicators to assist pathologists are desired in the diagnosis of such cases.

Our experimental results show that luteinized tumor cells in SST have abundant intracellular lipids, and Oil red O staining is obvious. Although microencapsulated stromal tumors and granulosa cell tumors have no intracellular lipids and Oil red O is negative, there can also be intracellular lipids in theca cell tumors. Meanwhile, there was no significant difference in immunohistochemical results between SSTs and other ovarian sexual cord stromal tumors except for TFE3 because SF-1, calretinin and inhibin were also expressed in these tumors. In our study, seven out of eight SST cases expressed luteinized cells with moderate-to-strong staining of TFE3, but it was negative in the ovarian granulosa, microcystic stromal tumor and thecoma/fibroma. Therefore, the positive expression of TFE3 immunohistochemistry is of great significance in the diagnosis and differential diagnosis of sclerosing stromal tumors [6, 8, 12, 15–17].

Moreover, FISH analysis revealed that the *TFE3* gene was not broken, indicating that there was no possibility of *TFE3* fusion with other genes. These results are consistent with those previously reported [22]. Combined with the results of Chamberlain et al. [18] and Schoolmeester et al. [19], these findings indicate that unlike ASPS, nuclear TFE3 expression in SST was not caused by genetic translocation, suggesting that other mechanisms may be involved. TFE3 is closely related to TFEB, both of which are part of the cell response to endoplasmic reticulum. Reticular stress causes its translocation to the nucleus. In cellular homeostasis mTOR phosphorylation prevents TFE3/TFEB activation and translocation into the nucleus. Under cellular stress/starvation, the decrease in mTOR phosphorylation leads to their nuclear translocation [38]. We speculate that the nuclear TFE3 expression in SST may be related to the phosphorylation of mTOR.

Tumors with nuclear TFE3 protein expression with or without gene fusion have some common morphological characteristics, such as abundant cytoplasm and obvious nucleoli [39]. In SST, we also found a type of cell with abundant cytoplasm and distinct nucleoli, which specifically expressed TFE3. These results indicate that the nuclear expression of TFE3 is related to cellular morphology, while there is no significant correlation with TFE3 gene abnormalities.

To explore the origin of TFE3-positive cells, we analyzed the expression of TFE3 in normal ovaries. To our knowledge, this is the first study to demonstrate that TFE3 is expressed in the theca interna cell nuclei of follicles but not in the granulosa cell layer, theca externa layer, or fibroblasts. During the development of ovarian follicles, the granulose cell layer is avascular, while the theca interna layer contains a rich vascular plexus. The nutrition of the egg is provided by the theca cell layer. According to the literature, TFE3 nuclear-positive epithelioid angioendothelioma with or without abnormal TFE3 gene expression is different from classic epithelioid angioendothelioma, which has obvious vascular formation [32]. Another study reported that TEF3 could affect the expression of VEGF [40] and Hirakawa T et al. reported that in immunohistochemical analysis, VEGF, bFGF and HGF were widely stained in SSTs [41]. We speculate that TFE3 will promote the formation of blood vessels after entering the nucleus, which can explain only the theca interna layer containing a rich vascular plexus and sclerosing stromal tumors with high vascularity.

In conclusion, our results show that TFE3 is expressed in the theca interna layer of normal follicles. Meanwhile, this study also suggests that the immunohistochemical detection of TFE3 is helpful for the diagnosis of difficult cases of sclerosing stromal tumors (e.g., cystic SST). Lipid-rich SST cells mimic the theca interna layer of normal ovaries and express TFE3 without disrupting the gene structure.

## Supplementary Information

**Additional file 1:** **Fig. S1.** The expression of TFE3 immunohistochemistry in 8 cases of sclerosing stromal tumors (x400). A-H correspond to cases 1-8 in Table 1, respectively. (A, D, F, and H). In some cases, the nucleus of TFE3 was strongly positive. (B). TFE3 showed moderate to strong nuclear positivity in one case of sclerosing stromal tumor. (C and G). TFE3 was moderately expressed. (E). TFE3 was weakly expressed.

**Additional file 2**: **Fig. S2.** External control of TFE3 immunohistochemistry. The negative control was prostate cancer. The positive control was TFE3 translocation-associated PEComa.

**Additional file 3**: **Table S1.** The expression of TFE3 in 8 cases of SSTs.

## Data Availability

The authors confirm that the data supporting the findings of this study are available within the article [and/or] its supplementary materials.

## Abbreviations

SST: Sclerosing Stromal tumors
TFE3: Transcription factor enhancer 3
MiTF: Microphthalmia-associated transcription factor
ASPS: Alveolar soft-part sarcoma
