# Supplementary figures and images for: TFE3 nuclear expression as a novel biomarker of ovarian sclerosing stromal tumors and associated with its histological morphology
Source: J Ovarian Res. 2023 Aug 1;16:152. doi: 10.1186/s13048-023-01241-y (PMC10394818; doi:10.1186/s13048-023-01241-y)

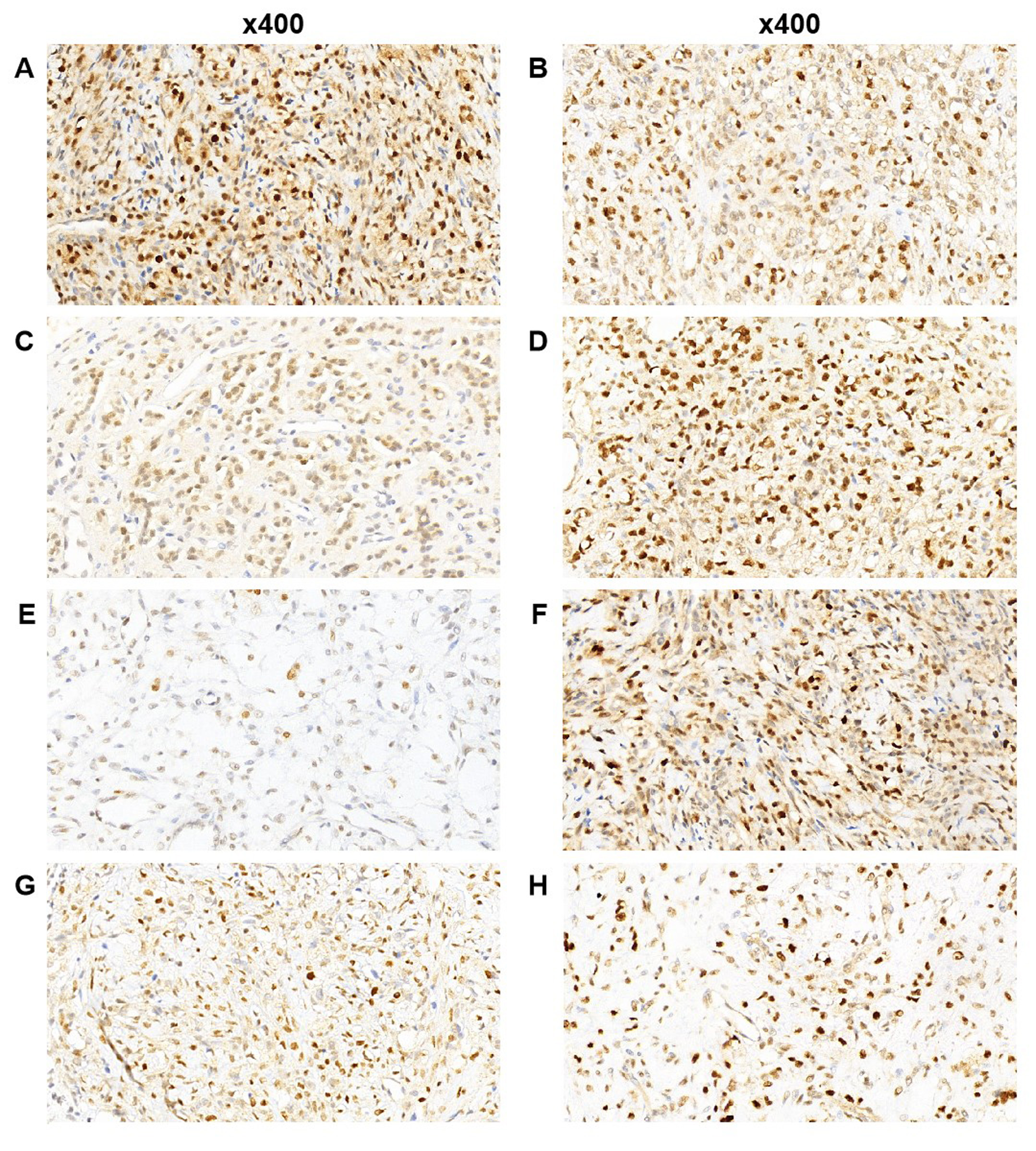

Supplement: Supplementary file 1 — Additional file 1: Fig. S1. The expression of TFE3 immunohistochemistry in 8 cases of sclerosing stromal tumors (x400). A-H correspond to cases 1-8 in Table 1, respectively. (A, D, F, and H). In some cases, the nucleus of TFE3 was strongly positive. (B). TFE3 showed moderate to strong nuclear positivity in one case of sclerosing stromal tumor. (C and G). TFE3 was moderately expressed. (E). TFE3 was weakly expressed. [file 13048_2023_1241_MOESM1_ESM.jpg]

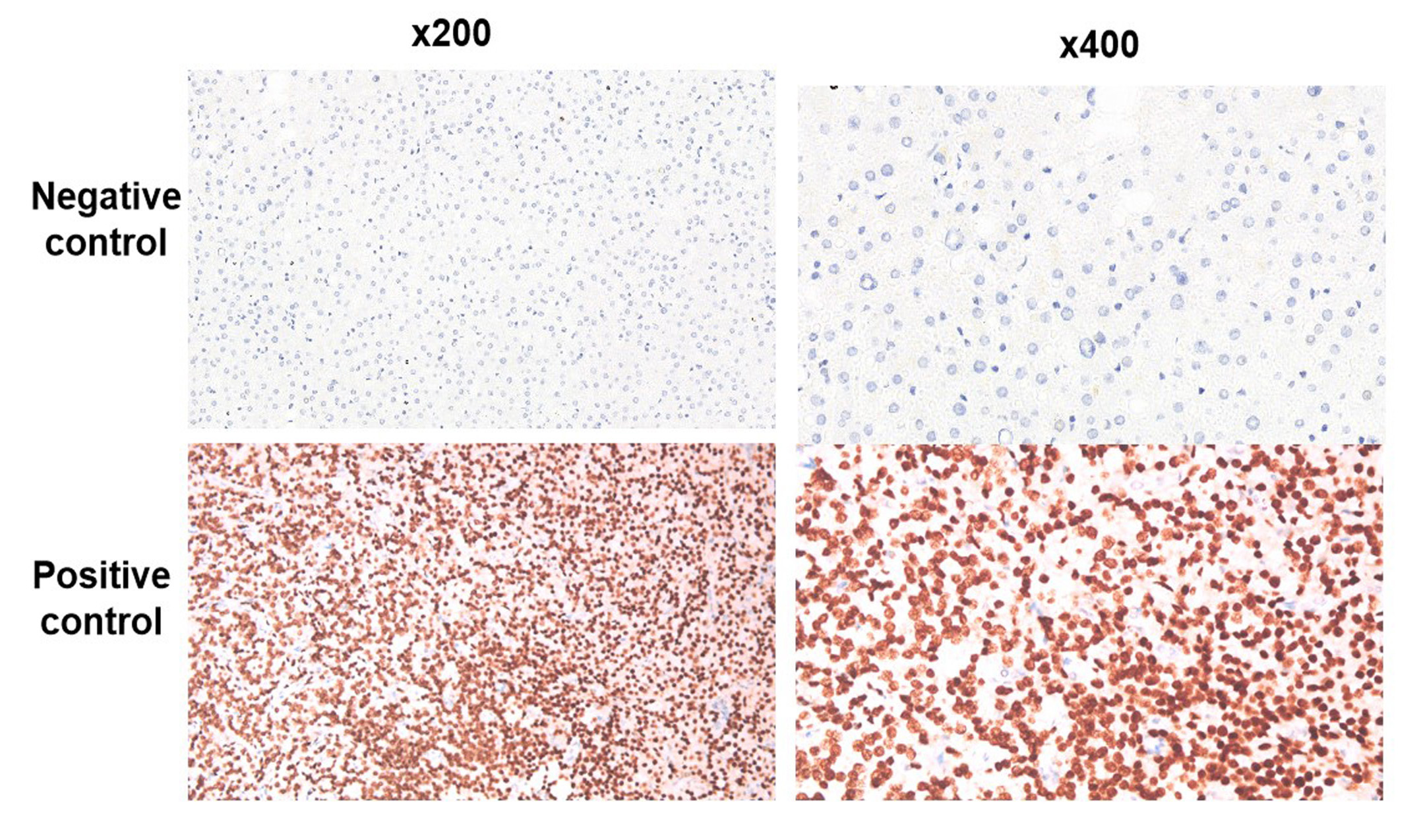

Supplement: Supplementary file 2 — Additional file 2: Fig. S2. External control of TFE3 immunohistochemistry. The negative control was prostate cancer. The positive control was TFE3 translocation-associated PEComa. [file 13048_2023_1241_MOESM2_ESM.jpg]
