# Supplementary material for: TFE3 nuclear expression as a novel biomarker of ovarian sclerosing stromal tumors and associated with its histological morphology
Source: J Ovarian Res. 2023 Aug 1;16:152. doi: 10.1186/s13048-023-01241-y (PMC10394818; doi:10.1186/s13048-023-01241-y)
Supplement: Supplementary file 3 — Additional file 3: Table S1. The expression of TFE3 in 8 cases of SSTs. [file 13048_2023_1241_MOESM3_ESM.docx]

Table S1.The expression of TFE3 in 8 cases of SSTs

| Patient with SSTs | intensity | percentage positivity(％) |
| --- | --- | --- |
| Case 1 | 3+ | 80 |
| Case 2 | 3+ | 40 |
|  | 2+ | 30 |
| Case 3 | 2+ | 80 |
| Case 4 | 3+ | 80 |
| Case 5 | 1+ | 20 |
| Case 6 | 3+ | 70 |
| Case 7 | 2+ | 80 |
| Case 8 | 3+ | 70% |
